# Concentrative Nucleoside Transporter, CNT, Results in Selective Toxicity of Toyocamycin against Candida albicans

**DOI:** 10.1128/spectrum.01138-22

**Published:** 2022-08-01

**Authors:** Yoshihiro Ojima, Naoki Yokota, Yuki Tanibata, Shinsuke Nerome, Masayuki Azuma

**Affiliations:** a Department of Chemistry and Bioengineering, Graduate School of Engineering, Osaka Metropolitan University, Osaka, Japan; University of Debrecen

**Keywords:** toyocamycin, *Candida albicans*, *Saccharomyces cerevisiae*, concentrative nucleoside transporter

## Abstract

Toyocamycin (TM) is an adenosine-analog antibiotic isolated from Streptomyces toyocaensis. It inhibits Candida albicans, several plant fungal pathogens, and human cells, but many fungi, including Saccharomyces cerevisiae, are much less susceptible to TM. Aiming to clarify why TM and its analogs tubercidin and 5-iodotubercidin are active against C. albicans but not S. cerevisiae, this study focused on the absence of purine nucleoside transport activity from S. cerevisiae. When the concentrative nucleoside transporter (CNT) of C. albicans was expressed in S. cerevisiae, the recombinant strain became sensitive to TM and its analogs. The expression of C. albicans purine nucleoside permease in S. cerevisiae did not result in sensitivity to TM. Clustered regularly interspaced short palindromic repeat-mediated disruption of CNT was performed in C. albicans. The CNTΔ strain of C. albicans became insensitive to TM and its analogs. These data suggest that the toxicity of TM and its analogs toward C. albicans results from their transport via CNT. Interestingly, S. cerevisiae also became sensitive to TM and its analogs if human CNT3 was introduced into cells. These findings enhance our understanding of the mechanisms of action of adenosine analogs toward *Candida* pathogens and human cells.

**IMPORTANCE** We investigated the mechanism of toxicity of TM and its analogs to C. albicans. Inspired by the effect of the copresence of TM and purine nucleosides on cell growth of C. albicans, we investigated the involvement of CNT in the toxicity mechanism by expressing CNT of C. albicans (CaCNT) in S. cerevisiae and deleting CaCNT in C. albicans. Our examinations clearly demonstrated that CaCNT is responsible for the toxicity of TM to C. albicans. S. cerevisiae expressing the human ortholog of CaCNT also became sensitive to TM and its analogs, and the order of effects of the TM analogs was a little different between CaCNT- and hCNT3-expressing S. cerevisiae. These findings are beneficial for an understanding of the mechanisms of action of adenosine analogs toward *Candida* pathogens and human cells and also the development of new antifungal drugs.

## INTRODUCTION

Toyocamycin (TM) is an antibiotic first isolated by Nishimura et al. (1) from Streptomyces toyocaensis. Its chemical structure was established without total synthesis (see Fig. S1 in the supplemental material) (2). Its analog tubercidin (Tbn) was later isolated from Streptomyces tubercidicus (3) and has an antibiotic spectrum similar to that of TM. The chemical structure of Tbn was established by Suzuki et al. (4) and Mizuno et al. (5) and shows a marked similarity to that of TM, differing only in the absence of a nitrile group at the 5-position. Meanwhile, 5-iodotubercidin (5-Itu) has an iodo group at the 5-position (6).

These compounds are all analogs of adenosine. They are highly toxic to human HeLa cells at low concentrations (<100 nM) (7), and they have somewhat specific anti-multiple myeloma activity (8). They also have a range of antiviral, antibacterial, and antifungal activities. Their modes of action are manifold, inhibiting many biological processes, including some glycolytic enzymes, RNA and DNA synthesis, and mRNA splicing of endoplasmic reticulum stress-induced XPB1 of human multiple myeloma cells (8) and human Rio1 kinase, an essential ribosome processing factor required for the proper maturation of the 40S ribosomal subunit (9). Candida albicans and several plant fungal pathogens are highly sensitive to these compounds; however, many fungi, including Saccharomyces cerevisiae, are much less susceptible (1).

In humans, C. albicans is an opportunistic pathogen that can cause either systemic or mucosal infection. Furthermore, this organism can progress to severe systemic invasion in immunocompromised patients, leading to life-threatening circumstances (10, 11). To find novel analogs of TM with fewer toxic side effects toward humans, it seems vital to clarify the mechanisms of action of TM and understand why it acts on C. albicans but not on *Saccharomyces* spp. Because TM is an adenosine analog, the difference in nucleoside transport between C. albicans and S. cerevisiae seems worth considering.

In contrast to S. cerevisiae, which usually lacks detectable purine nucleoside transport capability (12, 13), C. albicans possesses pyrimidine and purine nucleoside transporters (14, 15). The one nucleoside transport protein that is characterized so far in C. albicans is the purine nucleoside permease (NUP) (16). Characterized functionally in transformed S. cerevisiae, C. albicans NUP (CaNUP) transports purine nucleosides and thymidine, but not uridine. A concentrative nucleoside transporter (CNT) of C. albicans (CaCNT) has also been reported (17), but the S. cerevisiae genome encodes no CNT. CaCNT is an H^+^/nucleoside symporter of the major facilitator superfamily (MFS) of transporters and has 608 amino acid residues and 13 transmembrane segments. CaCNT expressed in Xenopus laevis oocytes mediated H^+^-coupled transport of uridine, adenosine, inosine, and guanosine, but not thymidine or cytidine (17). Tbn has also been transported by CNT in C. albicans (18), supporting a hypothesis that CNT is the critical factor making TM active toward C. albicans but not S. cerevisiae.

This study focused on the absence of purine nucleoside transport activity from S. cerevisiae to help clarify the mechanism of action of TM and its analogs toward C. albicans. First, we expressed CaNUP and CaCNT in S. cerevisiae W303-1A and examined the sensitivity of the resulting strains to TM. Furthermore, CNT disruption was conducted in C. albicans using the clustered regularly interspaced short palindromic repeat (CRISPR)-associated protein-9 (Cas9)-mediated system. Finally, human CNT3 (hCNT3) was introduced in S. cerevisiae to examine TM transport.

## RESULTS

### The antiyeast spectrum of TM.

**FIG 1:**
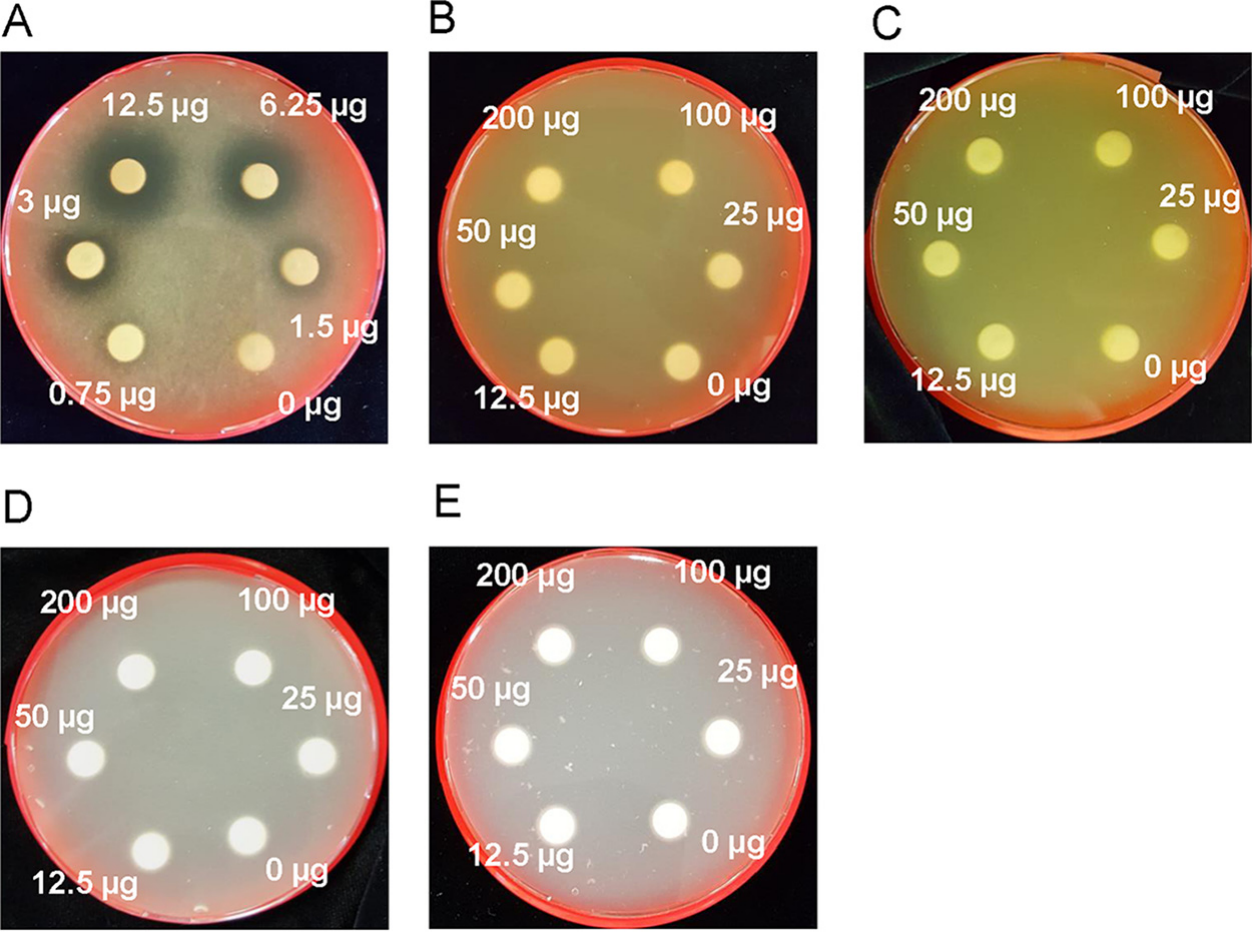
Photographs of plates seeded with yeast strains in toyocamycin (TM) disk diffusion tests. (A to C) YPD medium was used for Candida albicans SC5314 (A), Saccharomyces cerevisiae BY4741 (B), and *C. utilis* NBRC0988 (C). (D and E) YE medium was used for Schizosaccharomyces pombe JY746 (D), and Czapek medium was used for *Aureobasidium pullulans* NBRC4466 (E).

Disk diffusion tests were used to confirm the antiyeast spectrum of TM. Three strains—C. albicans, Candida utilis, and S. cerevisiae—were tested on yeast extract-peptone-dextrose (YPD) agar with disks containing 0 to 200 μg TM. Schizosaccharomyces pombe and Aureobasidium pullulans were tested on yeast extract (YE) and Czapek agar, respectively. As previously reported, C. albicans was sensitive to TM (Fig. 1). A growth inhibition circle formed with all the disks, even with the minimum amount of TM (0.75 μg/disk). A growth inhibition circle was never observed for the other yeast strains, even with the maximum amount of TM (200 μg/disk). Hence, these results support the specific growth repression of C. albicans by TM.

### Effect of the copresence of TM and purine nucleosides on cell growth of C. albicans.

**FIG 2:**
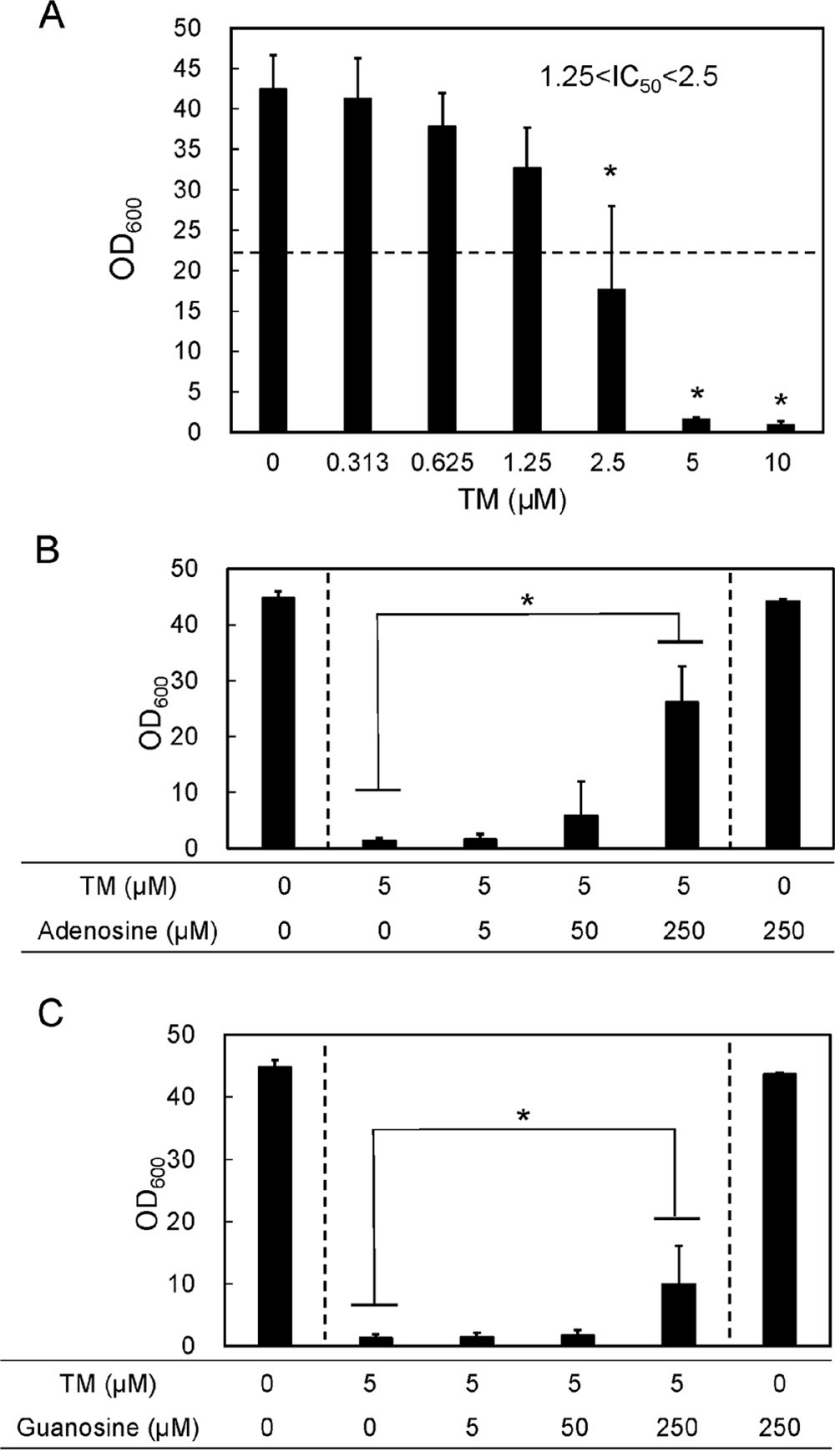
(A) Cell growth of C. albicans in the presence of TM and/or purine nucleosides. Effect of TM concentration on the OD_600_. (B and C) Effect of TM on the OD_600_ in the presence of adenosine (B) or guanosine (C). The dashed line indicates half the value under the control condition. The data were obtained from more than three independent experiments. Asterisks indicate statistically significant differences compared to control values (i.e., without TM for panel A) or between paired values (determined by analysis of variance [ANOVA] with the Tukey test [*P < *0.05]).

C. albicans cell growth was examined quantitatively in the presence of TM and/or a purine nucleoside (adenosine or guanosine). The cultures were grown under shaking conditions in test tubes, and optical density at 600 nm (OD_600_) values were measured at the end of the culture period. Figure 2A shows the dose-dependent effect of TM on the cell growth of C. albicans. TM did not significantly influence cell growth at ≤1.25 μM. At 2.5 μM TM, the OD_600_ decreased by 60% compared with that in the absence of TM. Therefore, the 50% inhibitory dose (IC_50_) of TM is estimated to be between 1.25 and 2.5 μM. More than 5 μM TM strongly suppressed the cell growth (to OD_600_ of ≤2).

Subsequently, cell growth was examined in the presence of both TM and adenosine or guanosine. Because TM is an adenosine analog, the copresence of these purine nucleosides may restore cell growth by a competitive effect on TM transport and/or TM metabolism in the cells. The TM-inhibited cell growth was restored in an adenosine dose-dependent manner (Fig. 2B). Without added adenosine, but in the presence of 5 μM TM, the final OD_600_ of the culture was 1.3 ± 0.6. This value slightly, but not significantly, increased when the adenosine concentration was 50 μM. However, at 250 μM adenosine, in the presence of 5 μM TM, the OD_600_ recovered to 26.2 ± 6.4, approximately 60% of that without addition of TM. As a control, in the absence of TM, the OD_600_ values for a culture with 250 μM adenosine was 43.8 ± 0.1, almost equal to that in the absence of TM and adenosine (44.8 ± 1.1), suggesting that adenosine itself did not promote the growth of C. albicans.

Meanwhile, the presence of guanosine slightly restored TM-inhibited cell growth (Fig. 2C). The cell growth was not restored by ≤50 μM guanosine, but it was restored by 250 μM guanosine, to 20% of the level in the absence of TM.

These results indicate that these purine nucleosides restored cell growth of C. albicans by a competitive effect on TM transport into the cells or TM action in the cells and that adenosine was more effective than guanosine. Considering that both adenosine and guanosine restored cell growth, we favor a competitive effect on TM transport as the mechanism.

### Introduction of C. albicans nucleoside transporters into S. cerevisiae.

S. cerevisiae does not have an adenosine transport system, whereas C. albicans does (17). To assess whether the growth inhibition of C. albicans, but not S. cerevisiae, by TM is related to nucleoside transport, two nucleoside transporters of C. albicans—NUP and CNT—were expressed in S. cerevisiae. First, the genes encoding these transporters were cloned into a plasmid expression vector, and the constructed vectors were introduced into S. cerevisiae strain W303-1A (an adenine auxotroph). This adenine auxotroph could not grow on minimal medium even in the presence of adenosine because S. cerevisiae lacks an adenosine transporter. However, if the recombinant CaNUP and CaCNT function in S. cerevisiae, the transformed strains are expected to grow with the complementation of adenine auxotrophy by adenosine.

**FIG 3:**
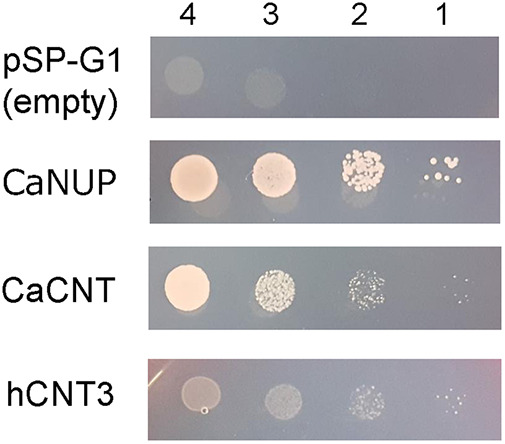
Colony formation of S. cerevisiae strain W303-1A transformed with C. albicans purine nucleoside permease (CaNUP), C. albicans concentrative nucleoside transporter (CaCNT), or human concentrative nucleoside transporter 3 (hCNT3) on YNB (–uracil) agar plates supplemented with 60 mg/L leucine, 20 mg/L histidine, 20 mg/L tryptophan, and 20 mg/L adenosine and incubated at 30°C for 2 days (or 3 days for hCNT3). The spots initially contained 10 μL of cell suspension with an OD_600_ of 1.0 (lane 4), 0.1 (lane 3), 0.01 (lane 2), or 0.001 (lane 1).

As shown in Fig. 3, strain W303-1A with empty plasmid (the negative control) did not form colonies on adenosine-supplemented YNB (–uracil) agar plates, indicating that strain W303-1A (as expected) did not transport adenosine. However, the S. cerevisiae strains with a plasmid carrying CaNUP or CaCNT did form colonies, suggesting that these transporters of C. albicans realize adenosine transport in S. cerevisiae. Conversion of imported adenosine to ATP and inosine in the cells enabled the adenine auxotroph S. cerevisiae W303-1A to grow on medium containing adenosine. Furthermore, hCNT3 was expressed in S. cerevisiae. The introduction of hCNT3 enabled colony formation on adenosine-supplemented YNB (–uracil) agar plates, suggesting that recombinant hCNT3 can also transport adenosine in S. cerevisiae.

### Sensitivity of recombinant S. cerevisiae strains to TM and its analogs.

The resultant S. cerevisiae strains were used for quantitative analysis of cell growth in the presence of TM. For the S. cerevisiae W303-1A strain carrying pSP-G1-CaNUP (W303-1A/CaNUP), the OD_600_ in the absence of TM was approximately 6.5. Moreover, cell growth was not suppressed even at a high concentration of TM (70 μM; data not shown). Because the functioning of CaNUP was confirmed by proliferation on adenosine-containing medium (Fig. 3), this result suggests that TM was not transported by CaNUP.

**FIG 4:**
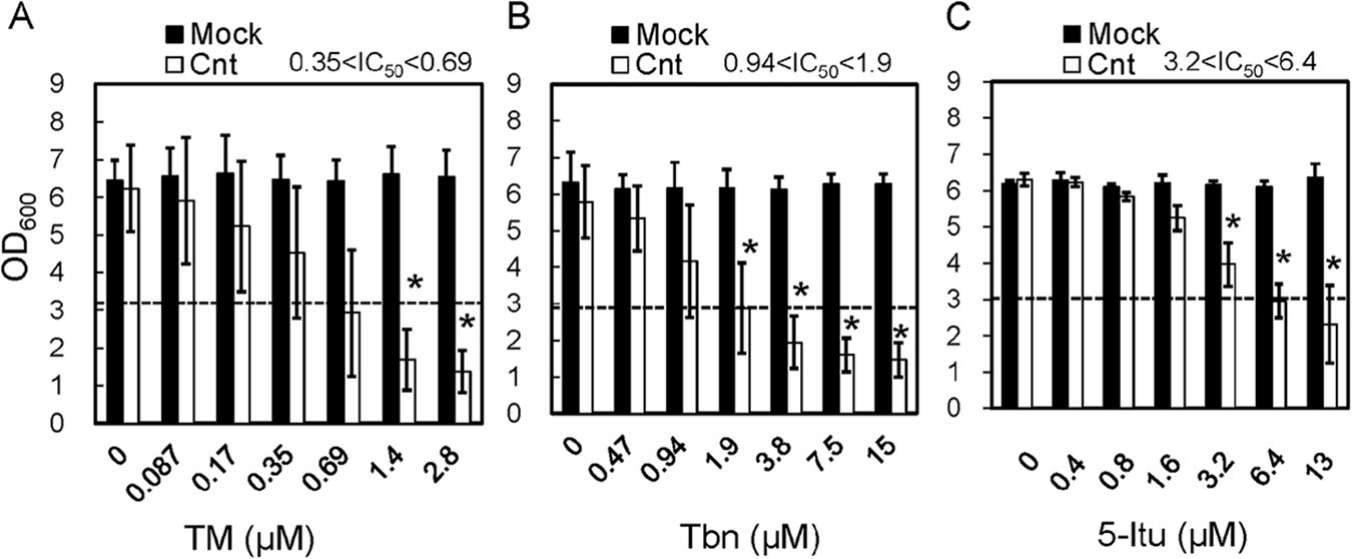
(A to C) Cell growth of S. cerevisiae strain W303-1A expressing CaCNT in YNB (–uracil) liquid medium with TM (A), tubercidin (Tbn) (B), or 5-iodotubercidin (5-Itu) (C). The dashed lines indicate half the value in the control condition. The data were obtained from more than three independent experiments. Asterisks indicate statistically significant differences compared to control values (i.e., without TM) determined by ANOVA with the Tukey test (*P < *0.05).

Next, strain W303-1A carrying the CaCNT expression plasmid was cultured in the presence of TM (Fig. 4A). The addition of TM did not significantly influence the cell growth of the negative control (carrying empty plasmid). However, the CaCNT-expressing strain showed TM dose-dependent repression of cell growth. At 2.8 μM TM, the OD_600_ was 1.4 ± 0.6, approximately 20% of that in the absence of TM. Thus, S. cerevisiae strain W303-1A became sensitive to TM by introduction of CaCNT, supporting the hypothesis that TM transport via CaCNT is a key mechanism in the selective toxicity of TM toward C. albicans.

The effects of Tbn and 5-Itu, analogs of TM, on the cell growth of CaCNT-expressing S. cerevisiae were also examined (Fig. 4B and C). The addition of either analog resulted in a dose-dependent decrease in cell growth. Therefore, these analogs are also transported by CaCNT. This result is consistent with the previous report that Tbn was transported by CaCNT (18). Comparing IC_50_ values, the order of effects of the TM analogs was TM > Tbn > 5-Itu.

**FIG 5:**
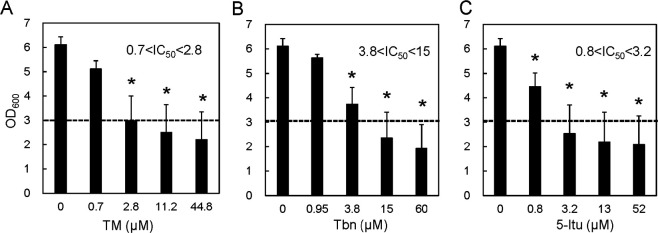
(A to C) Cell growth of S. cerevisiae strain W303-1A expressing hCNT3 in YNB (–uracil) liquid medium with TM (A), Tbn (B), and 5-Itu (C). The dashed line indicates half the value under the control condition. The data were obtained from more than three independent experiments. Asterisks indicate statistically significant differences compared to control values (i.e., without TM) determined by ANOVA with the Tukey test (*P < *0.05).

The cell growth of the hCNT3-expressing strain of S. cerevisiae was also examined in liquid cultures in the presence of TM (Fig. 5). The OD_600_ value of the cultures decreased with increasing TM concentration and reached approximately 2 at 44.8 μM TM. This result suggests that TM was transported by hCNT3 expressed in S. cerevisiae. Comparing the IC_50_ values, the order of effects of the TM analogs was TM ≈ 5-Itu > Tbn.

### Disruption of CNT from C. albicans using CRISPR-Cas9.

The experiments using recombinant S. cerevisiae suggested that the selective action of TM toward C. albicans could be explained by TM transport via CaCNT. Therefore, the gene encoding CNT in C. albicans was disrupted using the CRISPR-Cas9 system with plasmid pV1524, to generate C. albicans strain CNTΔ. Sequence analysis was conducted to confirm the CNT disruption in the genomic DNA of the resultant strain. As shown in Fig. S2, the total length of the native CNT-encoding gene is 1,824 bp. Sequence analysis revealed that a stop codon (TAA) was successfully inserted at 340 bp after the start codon of the CNT gene in-frame using the CRISPR-Cas9 system (Fig. S2), as designed in the repair template DNA. This insertion would be expected to cause the translation product to lose its function as a nucleoside transporter. Treatment with EcoRI completely digested the PCR-amplified target area (data not shown), suggesting that homozygous recombination was achieved in the genome of diploid C. albicans. This result is consistent with the report that the CRISPR system adopted in this study achieved homozygous mutation of a target gene in one transformation (19).

**FIG 6:**
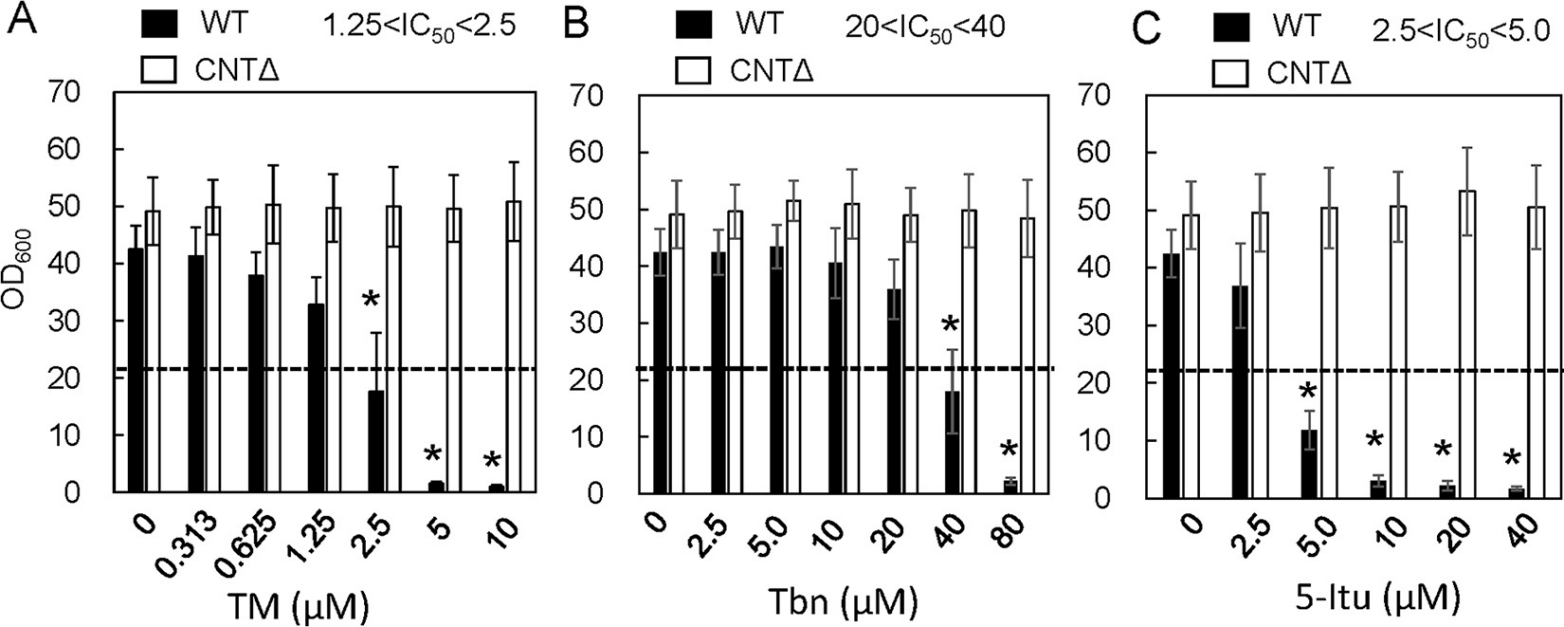
(A to C) Cell growth of C. albicans strain CNTΔ in YNBP liquid medium with TM (A), Tbn (B), and 5-Itu (C). The dashed line indicates half the value under the control condition. The data were obtained from more than three independent experiments. Asterisks indicate statistically significant differences compared to control values (i.e., without TM) determined by ANOVA with the Tukey test (*P < *0.05).

The constructed C. albicans CNTΔ strain was exposed to TM and its analogs. As expected, the CNTΔ strain was not sensitive to TM (Fig. 6). Cell growth of the wild-type strain was poor at ≥5 μM TM, but no growth repression was observed for the CNTΔ strain at up to 10 μM TM. This result strongly supports that the action of TM toward C. albicans results from TM transport via the CNT. A similar tendency was observed for the TM analogs Tbn and 5-Itu; the growth of the CNTΔ strain did not decrease even at concentrations at which the wild-type strain could hardly grow.

## DISCUSSION

TM is an antibiotic isolated from *S. toyocaensis* with a specific antibiotic spectrum (1). In this study, we examined the effect of TM on several types of yeast. It was confirmed that TM did not inhibit the cell growth of *C. utilis*, S. cerevisiae, S. pombe, or *A. pullulans*. To help clarify the mechanism of action of TM and its analogs toward C. albicans, C. albicans cell growth was examined quantitatively in the presence of TM and/or purine nucleosides (adenosine or guanosine). Both purine nucleosides restored the cell growth of C. albicans in the presence of TM, and adenosine was more effective than guanosine. This difference in cell growth recovery is explained by the finding in the previous report that the CaCNT has a smaller *K_m_* value for adenosine than guanosine (17). From these results, we hypothesized that the mechanism by which the presence of purine nucleosides lowered the toxicity of TM mainly involved inhibition of TM transport into the cells.

**FIG 7:**
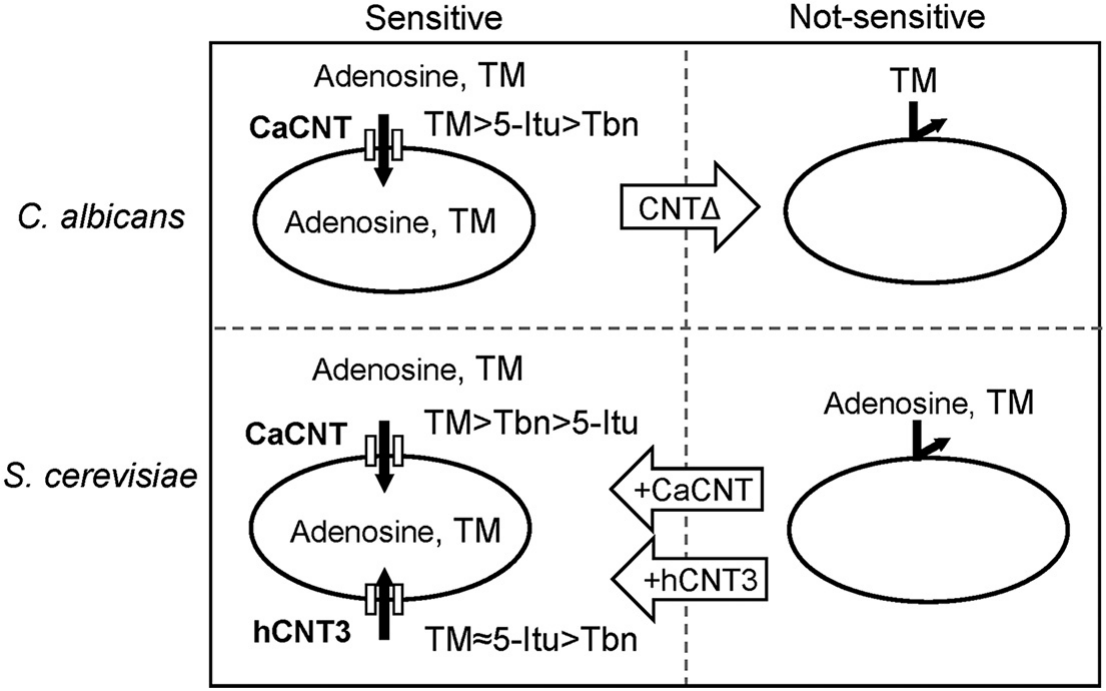
Model of the mechanism underlying the specificity of TM and its analogs toward C. albicans.

S. cerevisiae does not have an adenosine transport system, whereas C. albicans does (17). TM is toxic toward C. albicans but not S. cerevisiae. Thus, to analyze the mechanism of growth inhibition of C. albicans by TM, two nucleoside transporters of C. albicans were expressed in S. cerevisiae. Two nucleoside transporters have been reported in C. albicans: (i) the purine nucleoside permease CaNUP, which transports adenosine and guanosine (16), and (ii) an Na^+^-independent nucleoside transporter, CaCNT, which transports adenosine, guanosine, uridine, inosine, and Tbn by electrogenic, H^+^-dependent transport (17, 18). For this analysis, we selected S. cerevisiae strain W303-1A (an adenine auxotroph), which does not grow in minimal medium containing adenosine (20). Because adenosine complements the adenine auxotrophy after being taken up into cells, it is possible to confirm the function of recombinant nucleoside transporters by evaluating the cell growth of strain W303-1A. CaNUP and CaCNT were found to function as adenosine transporters in S. cerevisiae strain W303-1A (Fig. 7). CaCNT-expressing S. cerevisiae W303-1A became sensitive to TM and its analogs. However, the expression of CaNUP in S. cerevisiae did not result in sensitivity. These results suggest that TM and its analogs were taken up by CNT, but not by NUP, reflecting the broad spectrum of nucleoside selectivity of CNT compared with that of NUP (17).

CRISPR-Cas9-mediated CaCNT disruption was conducted in C. albicans to support the hypothesis that TM and analogs were transported by CNT. The CNTΔ strain of C. albicans was insensitive to TM and its analogs, suggesting that they are specifically transported through CNT and that CNT is the only transporter for these compounds in C. albicans.

S. cerevisiae strain W303-1A also became sensitive to TM and analogs when human CNT3 was introduced into the cells. There are three CNTs in humans (hCNT1, hCNT2, and hCNT3). hCNT3 has higher homology to CaCNT than the other two human CNTs (17). Moreover, hCNT3 has the broadest spectrum for transportation of nucleosides and analogs and has been reported to transport pyridine, purine nucleosides, and inosine (17). In recombinant S. cerevisiae expressing hCNT3, cell growth was repressed by the transport of TM and its analogs. This result is consistent with the previous report that TM and its analogs suppressed the growth of HeLa cells (7). As far as we know, this is the first report showing that human CNTs work in yeast. Interestingly, the order of effects of Tbn and 5-Itu changed between S. cerevisiae expressing CaCNT and hCNT3. It is thought to be due to the difference in the substrate specificity of these transporters. Although we did not find a great difference in transportation of the TM analogs examined in this study, this difference in the substrate specificity may be a hint for the search for compounds specifically transported by CaCNT.

### Conclusions.

This study established that CaCNT-expressing S. cerevisiae became sensitive to TM and its analogs. In contrast, NUP expression in S. cerevisiae did not result in sensitivity. S. cerevisiae also became sensitive to TM and its analogs on introduction of hCNT3. Meanwhile, a CNTΔ strain of C. albicans became insensitive to TM and its analogs. These findings suggest that the action of TM and analogs toward C. albicans is dependent on the transport of those drugs via the CNT.

## MATERIALS AND METHODS

### Yeast strains.

Table 1 summarizes the yeast strains and plasmids used in this study. The main target strains were C. albicans SC5314 (21) and S. cerevisiae BY4741 (22) and W303-1A (20). Additionally, *C. utilis* NBRC0988, S. pombe JY476 (23), and *A. pullulans* NBRC4466 were used.

**TABLE 1:** Yeast strains and plasmids used in this study

Strains or plasmids	Genotype	Reference or source
Strains		
C. albicans SC5314	*Ura3*Δ::*imm434*/*ura3*Δ::*imm434*	21
CNTΔ	SC5314CNTΔ	This study
S. cerevisiae BY4741	Mating type a (MATa) his3Δ1 leu2Δ0 met15Δ0 ura3Δ0	22
S. cerevisiae W303-1A	Mating type a (MATa) ade2-1 ura3-1 his3-11 trp1-1 leu2-3 leu2-112 can1-100	20
*C. utilis* NBRC0988	Wild type	NBRC0988
S. pombe JY746	h+ ade6-M210 leu1 ura4-D18	23
*A. pullulans* NBRC4466	Wild type	NBRC4466
Plasmids		
pSP-G1	Yeast expression plasmid derived from pESC-URA	24
pSP-G1-CaNUP	pSP-G1 carrying CaNUP	This study
pSP-G1-CaCNT	pSP-G1 carrying CaCNT	This study
pSP-G1-hCNT3	pSP-G1 carrying hCNT3	This study
pV1524	Empty vector (NAT, CaCas9) suitable for CRISPR	25
pV1524-CaCNT	pV1524 carrying gRNA for CaCNT	This study

Plasmid DNA was prepared using Escherichia coli strain JM109 or DH5α. The high-copy-number plasmid pSP-G1 with constant expression from the PGK1 promoter was used for expressing target genes (CaNUP, CaCNT, and hCNT3). pSP-G1 was provided by NBRP, Japan (24). pV1524 was used for CRISPR-mediated CNT disruption in C. albicans (25) and was a gift from Gerald Fink (Addgene plasmid no. 111431; http://n2t.net/addgene:111431; RRID: Addgene_111431).

### Disk diffusion tests.

Yeast strains were precultured in test tubes at 30°C overnight with shaking. C. albicans, S. cerevisiae, and *C. utilis* were cultured in liquid YPD medium (1% [wt/vol] yeast extract, 2% [wt/vol] peptone, and 2% [wt/vol] glucose), S. pombe was cultured in liquid YE medium (0.5% [wt/vol] yeast extract and 3% [wt/vol] glucose), and *A. pullulans* was cultured in Czapek medium (0.1% [wt/vol] K_2_HPO_4_, 0.05% [wt/vol] MgSO_4_·7H_2_O, 0.001% [wt/vol] FeSO_4_·7H_2_O, 0.2% [wt/vol] NaNO_3_, 0.05% [wt/vol] KCl, and 4% [wt/vol] d-fructose). The cell suspension of each yeast strain was inoculated into fresh medium containing 0.5% agar in a petri dish at an OD_600_ of 0.25. A paper disk (ADVANTEC; 8-mm thick) impregnated with different concentrations of TM was placed on the agar medium, and the dishes were incubated at 30°C. In controls, a paper disk impregnated with dimethyl sulfoxide was used. The antibacterial spectrum of the drug was evaluated by observing whether a growth inhibition circle formed for each strain.

### Construction of plasmids and recombinant S. cerevisiae strains.

For constructing expression plasmids, the genes encoding C. albicans NUP and CNT (CaNUP and CaCNT) were amplified from genomic DNA of C. albicans using specific primers (Table S1). hCNT3 was amplified from artificially synthesized DNA whose sequence was optimized for expression in S. cerevisiae (Fig. S3). The fragments encoding CaNUP and CaCNT were treated with HindIII and BamHI and cloned into pSP-G1 (24). The hCNT3 fragment was treated with SmaI and KpnI and cloned into pSP-G1. DNA sequencing confirmed the insertions, and the constructed plasmids were named pSP-G1-CaNUP, pSP-G1-CaCNT, and pSP-G1-hCNT3, respectively. These plasmids were introduced into S. cerevisiae strain W303-1A by lithium acetate transformation, and the resultant strains were named in the style W303-1A/CaNUP and so on.

Colony-forming efficiency was examined using a solid-medium assay to examine the function of the introduced gene. In brief, cells of each strain were precultured in 4 mL of YNB (–uracil) medium (0.17% [wt/vol] yeast nitrogen base without ammonium sulfate, 0.5% [wt/vol] ammonium sulfate, 2.0% [wt/vol] glucose, 60 mg/L leucine, 20 mg/L histidine, 20 mg/L tryptophan, and 20 mg/L adenine) at 30°C overnight. The cells were collected by centrifugation (3,000 × *g*, 5 min) and then washed with phosphate-buffered saline (PBS). Diluted cell suspension (10 μL; OD_600_ of 0.001, 0.01, 0.1, or 1.0) was spotted onto solid YNB (–uracil) medium supplemented with 20 mg/L adenosine instead of adenine, and cells were incubated at 30°C for 2 days (3 days for hCNT3).

### Disruption of CNT from C. albicans using CRISPR.

A mutant strain of C. albicans with disrupted CNT was constructed from C. albicans strain SC5314 by genome editing using a single-plasmid CRISPR-Cas9 system (pV1524) (25, 26, 27). Briefly, the single guide RNA (sgRNA) was designed using CRISPRdirect (https://crispr.dbcls.jp/), and the target sequence 5′-GGTTGATTCCAACGGTTATT-3′ was determined at 20 mers upstream of the protospacer-adjacent motif (PAM) sequence (TGG) in the CNT gene. The pair of oligonucleotides (pV1524-sgRNA-CaCNT_F and R in Table S1) were annealed and cloned into the BsmBI-v2 site of pV1524. The insertion was confirmed by DNA sequencing, and the resultant plasmid pV1524-CaCNT was linearized by treatment with KpnI and SacI to increase the recombination efficiency. The repair template DNA was prepared by amplification using the primer set in Table S1, including a stop codon and an EcoRI site.

C. albicans SC5314 was precultured in 4 mL of YPD medium at 30°C with shaking overnight. The cells were inoculated into fresh YPD medium at an OD_600_ of 0.25 and cultured for 4 h at 30°C with shaking. The cells were harvested by centrifugation at 3,000 × *g* for 5 min and washed with PBS (pH 7.5). The cells were resuspended in 1.5 mL of LiAc-sol (100 mM LiAc, 10 mM Tris-HCl, 1 mM EDTA·Na_2_). The resultant solution (100 μL) was mixed with 600 μL LiAc-sol (50% polyethylene glycol 4000), 10 μg linearized pV1524-CaCNT, 6 μg repair template DNA, and 40 μL carrier DNA (salmon sperm, sonicated) and incubated for 24 h at 30°C. After incubation, the sample was heat-shocked at 44°C for 25 min. The cells were harvested by centrifugation at 3,000 × *g* for 5 min and washed with 1 mL of YPD medium. The cells were resuspended in 200 μL of YPD medium and incubated for 90 min at 30°C. A 100-μL sample was inoculated onto a YPD agar plate containing 200 μg/mL nourseothricin and incubated for 2 days. Genomic DNA was extracted from the colonies, and sequence analysis was performed to confirm CNT disruption. The resulting strain was named C. albicans CNTΔ (Table 1).

### Cell growth assays.

C. albicans SC5314 was precultured in YNBP medium (0.17% Difco yeast nitrogen base without amino acids, 0.5% ammonium sulfate, 2% peptone, 2% glucose, and 20 mg/L uracil). S. cerevisiae W303-1A-carrying plasmids were precultured in YNB (–uracil) medium supplemented with 60 mg/L leucine, 20 mg/L histidine, 20 mg/L tryptophan, and 20 mg/L adenine. All test cultures with shaking overnight were inoculated into 4 mL of each fresh medium in a test tube to an OD_600_ of 0.25. Adenosine, guanosine, TM, Tbn, and 5-Itu were added when necessary. The cells were cultured with shaking at 140 rpm at 30°C for 24 h. Cell growth was measured by OD_600_ values.

### Statistical analysis.

Each result is presented as the mean ± standard deviation from more than three independent experiments.
