# Supplementary material for: Concentrative Nucleoside Transporter, CNT, Results in Selective Toxicity of Toyocamycin against Candida albicans
Source: Microbiol Spectr. 2022 Aug 1;10(4):e01138-22. doi: 10.1128/spectrum.01138-22 (PMC9431476; doi:10.1128/spectrum.01138-22)
Supplement: Supplemental file 1 — Supplemental material. Download spectrum.01138-22-s0001.pdf, PDF file, 0.1 MB [file spectrum.01138-22-s0001.pdf]

Supplementary Table S1 Primers used in this study.

| Primer | Description |
|---|---|
| Expression vectors | |
| pSP-G1-CaNUP_F | 5'- AAAGGATCCATGAAGTTATCCACTTTATTTACATTA -3' |
| pSP-G1-CaNUP_R | 5'- AAACTCGAGTTAGTGGTGGTGGTGGTGGTGAGCAGTATCAAATGATTCTTTACC -3' |
| pSP-G1-CaCNT_F | 5'- AAAGGATCCATGGTTTCTCCGTCCACAGATAAAG -3' |
| pSP-G1-CaCNT_R | 5'- AAACTCGAGCTAGTGGTGGTGGTGGTGGTGGTTAATGTGGAAAGTGTTTAAATCATG -3' |
| pSP-G1-hCNT3_F | 5'- AAACCCGGGATGGAACTGCGTTCAACC -3' |
| pSP-G1-hCNT3_R | 5'- AAAGGTACCTTAGTGGTGGTGGTGGTGGTGGAATGTATTGCTAATGCCATTG -3' |
| CRISPR-Cas9 system | |
| pV1524-sgRNA-CaCNT_F | 5'- ATTTGGGTTGATTCCAACGGTTATTG -3' |
| pV1524-sgRNA-CaCNT_R | 5'- CCCAACTAAGGTTGCCAATAACAAAA -3' |
| Repair Template DNA_F | 5'- ATTCAACCTAAACATAGACATCAATGGTTGATTCCAACGGTTATTTAAGAATTCGGTATG -3' |
| Repair Template DNA_R | 5'- TAAAATTTTTATGTGCCAAGTGATCAATCTCACCATAATCATACCGAATTCTTAAATAAC -3' |

Supplementary Fig. S1

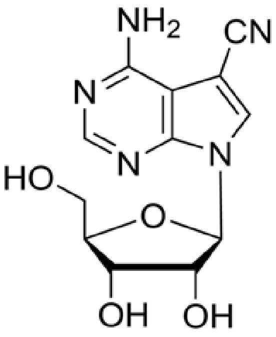

Toyocamycin (TM)

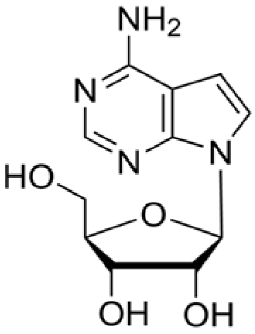

Tubercidin (Tbn)

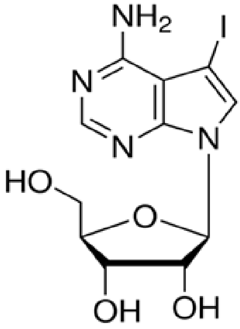

5-iodotubercidin (5-Itu)

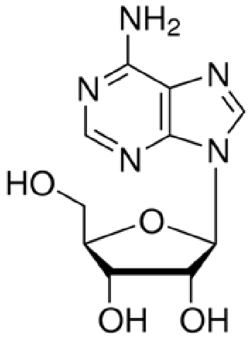

Adenosine

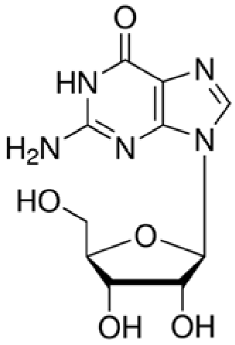

Guanosine

Supplementary Fig. S2

>CaCNT
ATGGTTTCTCCGTCCACAGATAAAGCACCATCCATTGTAGAGTTGACTCCAGAAACATATCAACAAGATGTTTCCTCAGATTCAA
TCGATTTGGAATCAGGTACAAAAAAGCTCCAAAATGAATCAGCTTTGCAATCACACGAGTATACTACCAATATCGATGAAACTTC
ATCAAGCAACACTGCATCCAAGTTGACTTATATCCAAAAATTGAAAATAAGATTTCCTTATTACAGATTAGCAATTGACATTTTT
ATCGGTTGCTTTTTCACTGCATGGTGGTTATCTATAGTCATTCAACCTAAACATAGACATCAATGGTTGATTCCAACGGTTATTT
GGGGTATGATTATGGTGAGATTGATCACTTGGCACATAAAAATTTTACCATGGTTATTAAACAAAGTCAAAATTGTTTGGGATTT
CTTTACTGGTTATGTGTATAAAGTTTTATCAAAAAAATATCAAAGATTAATCACTGGTGCTGTGATTACTGTTGGTGTTATTTTA
TTAGGTACATTTGTTCCTTCAGAAACTGAATATTCCAAAAGGAAAGATAGAGCCATCTCCTTTTTCGGTTGTATTGTTGCCATAT
TCTTATTGTTTGTCACTTCAAAAGCTCCTTCGAAAATTAATTGGAATGCGGTTATTGGCGGTATGTTGATGCAATTTATTATTGC
ATTATTTGTTTTGAGAACTAAGTGTGGGTACGATGTATTTAATTTCATTTCCACTTTGGCAAGAGAATTATTGGGTTTCGCCAAA
GATGGGGTGGCATTTTTAACTAATAAAGATGTCTCTCAATTAGGAATGTTCTTTTTCACCGTGTTACCTTCAGTGGCTTTTTTCG
TGGCGTTCATTCATATTTGGTATTATTTCGGTGTTATTCAATGGGCCATTAGAAAATTTGCTTACTTTTTCTTTTGGACATTAAG
AGTTTCTGGTGCTGAAGCCATTACAGCTGCTGCCTCTCCGTTTATCGGTATTGGTGAAAGTGCCATTTTAATTAAAGATTTGATG
CCATATTTGACTAAAGCAGAATTACATCAAATCATGACTTCAGGGTTTAGTACCATTAGTGGTGCTGTTCTTGTTGGTTATATTG
GTCTTGGTCTTAATCCACAAGCTTTGGTTAGTAGTTGTGTCATGTCAATTCCTGCATCTCTTGCAGTATCAAAATTAAGATACCC
TGAACTTGAAAACCCAATCTCAAGTGGTACAGTAATGATTCCAAAAGTTGAAGACCCTGAAGAAGCAAGGGAAAAATCAAAAGAT
GAACCTCAAAATGTCTTGCAAGCATTTTCAAATGGGGCCACTTTAGGGTTGAGAATTGCCGGGACAATGATGATTCAGTGTATGT
GTATTATTGGACTTGTTGCCTTATGCAATGGTATTTTAACATGGTTTGGTAACTATTGGAACATTGATCATTTGACTTTGGAATT
GATGCTTTCCTACATTTTTTACCCAATTGGATTCTTGTTGGGTACTCCGCGTAATGAAATTTTGCTTGTTAGTAAATTGATTGCT
TATAAATTCATTCAAAATGAATATGTTGCTTATAATTTGTTAACAAATGAAGCTCCTTATAATGAAATGTCTAAAAGAGGAACAT
TAATTGCCACCTATGCTTGTTGTGGGTTTGCCAATTTAGGTTCTTTGGGTATTACTTTGGGTGTTTTGAATACATTGACAAACAA
TTCTAGAGCCAAAGATATTTCTTCAAGTATTATATCTGCTTTGTTCTGTGGTGCCATTGCCACTATGTTATCTGCTGCCATTGCT
GGTATGGTTATGCATGATTTAAACACTTTCCACATTAACTAG (1827 bp)

>CNTΔ (Stop codon TAA was inserted at 340~342 bp in flame)
ATGGTTTCTCCGTCCACAGATAAAGCACCATCCATTGTAGAGTTGACTCCAGAAACATATCAACAAGATGTTTCCTCAGATTCAA
TCGATTTGGAATCAGGTACAAAAAAGCTCCAAAATGAATCAGCTTTGCAATCACACGAGTATACTACCAATATCGATGAAACTTC
ATCAAGCAACACTGCATCCAAGTTGACTTATATCCAAAAATTGAAAATAAGATTTCCTTATTACAGATTAGCAATTGACATTTTT
ATCGGTTGCTTTTTCACTGCATGGTGGTTATCTATAGTCATTCAACCTAAACATAGACATCAATGGTTGATTCCAACGGTTATT
TAAGAATTCGGTATGATTATGGTGAGATTGATCACTTGGCACATAAAAATTTTACCATGGTTATTAAACAAAGTCAAAATTGTT
TGGGATTTCTTTACTGGTTATGTGTATAAAGTTTTATCAAAAAAATATCAAAGATTAATCACTGGTGCTGTGATTACTGTTGGTG
TTATTTTATTAGGTACATTTGTTCCTTCAGAAACTGAATATTCCAAAAGGAAAGATAGAGCCATCTCCTTTTTCGGTTGTATTGT
TGCCATATTCTTATTGTTTGTCACTTCAAAAGCTCCTTCGAAAATTAATTGGAATGCGGTTATTGGCGGTATGTTGATGCAATTT
ATTATTGCATTATTTGTTTTGAGAACTAAGTGTGGGTACGATGTATTTAATTTCATTTCCACTTTGGCAAGAGAATTATTGGGTT
TCGCCAAAGATGGGGTGGCATTTTTAACTAATAAAGATGTCTCTCAATTAGGAATGTTCTTTTTCACCGTGTTACCTTCAGTGGC
TTTTTTCGTGGCGTTCATTCATATTTGGTATTATTTCGGTGTTATTCAATGGGCCATTAGAAAATTTGCTTACTTTTTCTTTTGG
ACATTAAGAGTTTCTGGTGCTGAAGCCATTACAGCTGCTGCCTCTCCGTTTATCGGTATTGGTGAAAGTGCCATTTTAATTAAAG
ATTTGATGCCATATTTGACTAAAGCAG·····················

Supplementary Fig. S3

>Sequence of hCNT3 codon optimized for *S. cerevisiae*（2076bp）

ATGGAACTGCGTTCAACCGCCGCACCTAGAGCAGAGGGGTATTCGAATGTTGGCTTTCAGAATGAGGAGAATTTCCTCGA
AAATGAAAATACTTCAGGCAATAACAGTATTCGCTCGAGAGCCGTACAATCTAGAGAACACACCAATACTAAACAGGATG
AAGAACAGGTGACTGTTGAGCAAGATAGTCCTAGGAATAGAGAACATATGGAAGACGATGACGAAGAAATGCAACAAAAG
GGTTGTCTGGAAAGACGATATGACACAGTCTGTGGTTTTTGTCGTAAACACAAGACAACCTTAAGGCACATAATCTGGGG
CATCCTACTGGCTGGATATCTAGTGATGGTGATTTCAGCTTGCGTTCTTAACTTCCATAGAGCTTTACCGCTTTTTGTCA
TTACTGTAGCTGCAATTTTCTTCGTTGTGTGGGATCACTTAATGGCCAAATACGAGCATCGTATTGACGAAATGCTTTCT
CCTGGAAGAAGATTGCTAAATTCGCATTGGTTTTGGTTGAAATGGGTTATCTGGTCCTCCTTAGTCTTAGCGGTCATCTT
TTGGTTGGCTTTTGATACTGCGAAACTTGGGCAACAGCAACTCGTCTCATTTGGTGGCTTAATCATGTATATAGTGCTGC
TTTTTTTGTTCTCTAAGTATCCAACCAGAGTATATTGGAGGCCAGTACTATGGGGTATAGGTCTTCAATTTTTGCTTGGC
TTGTTGATCTTACGCACGGATCCAGGCTTTATAGCTTTTGATTGGTTGGGTAGACAAGTGCAGACATTCTTAGAATACAC
TGATGCAGGAGCATCATTCGTTTTTGGAGAGAAATACAAGGATCACTTCTTCGCTTTTAAGGTTTTACCGATTGTCGTAT
TTTTCTCAACGGTCATGTCTATGCTATACTATCTAGGTTTGATGCAATGGATTATCAGAAAGGTTGGTTGGATTATGTTG
GTTACTACTGGATCTAGTCCCATAGAATCTGTTGTTGCCAGTGGTAACATTTTCGTTGGGCAAACAGAGTCACCATTATT
GGTTAGACCTTACTTACCCTATATCACGAAGAGTGAATTACATGCCATCATGACCGCTGGTTTCAGCACAATTGCAGGTT
CAGTTTTAGGTGCCTACATTAGCTTCGGAGTACCATCATCTCACTTGTTAACTGCATCCGTAATGTCTGCTCCTGCGTCA
TTAGCAGCAGCAAAGCTTTTTTGGCCTGAAACCGAAAAACCCAAAATTACACTGAAAAATGCGATGAAGATGGAGTCTGG
TGATTCCGGTAACTTGCTAGAAGCAGCTACACAAGGCGCATCCTCTTCCATAAGCCTAGTAGCCAATATAGCCGTTAACC
TCATTGCCTTTTTAGCATTGCTATCCTTTATGAACAGTGCTCTTTCCTGGTTTGGTAACATGTTTGATTATCCGCAATTG
TCTTTCGAATTGATATGCTCATACATATTCATGCCCTTTTCGTTTATGATGGGTGTGGAATGGCAAGACTCTTTCATGGT
CGCTAGATTGATTGGATACAAGACCTTTTTCAACGAGTTTGTAGCCTATGAACATTTATCTAAGTGGATACATCTTAGGA
AAGAAGGAGGTCCAAAATTTGTCAATGGTGTGCAACAGTATATTTCCATACGATCGGAAATCATTGCTACATATGCTTTG
TGTGGGTTTGCTAATATAGGGAGTTTAGGCATCGTTATTGGTGGCCTGACATCGATGGCTCCTTCTCGTAAACGGGATAT
TGCCTCTGGAGCTGTTAGGGCTCTAATTGCTGGAACTGTGGCGTGCTTCATGACTGCATGTATCGCAGGTATCTTAAGCT
CAACACCAGTAGACATTAACTGTCATCATGTGTTGGAGAACGCGTTTAACAGTACTTTTCCAGGTAATACGACCAAAGTT
ATAGCTTGCTGTCAAAGCCTGCTCTCATCTACAGTCGCCAAAGGACCTGGTGAAGTTATTCCAGGTGGGAACCATAGTTT
ATACTCCTTGAAAGGTTGTTGTACGCTGTTGAATCCAAGCACTTTTAATTGCAATGGCATTAGCAATACATTCTAA
